# Supplementary material for: GLI transcriptional repression is inert prior to Hedgehog pathway activation
Source: Nat Commun. 2022 Feb 10;13:808. doi: 10.1038/s41467-022-28485-4 (PMC8831537; doi:10.1038/s41467-022-28485-4)
Supplement: Supplementary file 1 — Supplementary Information [file 41467_2022_28485_MOESM1_ESM.pdf]

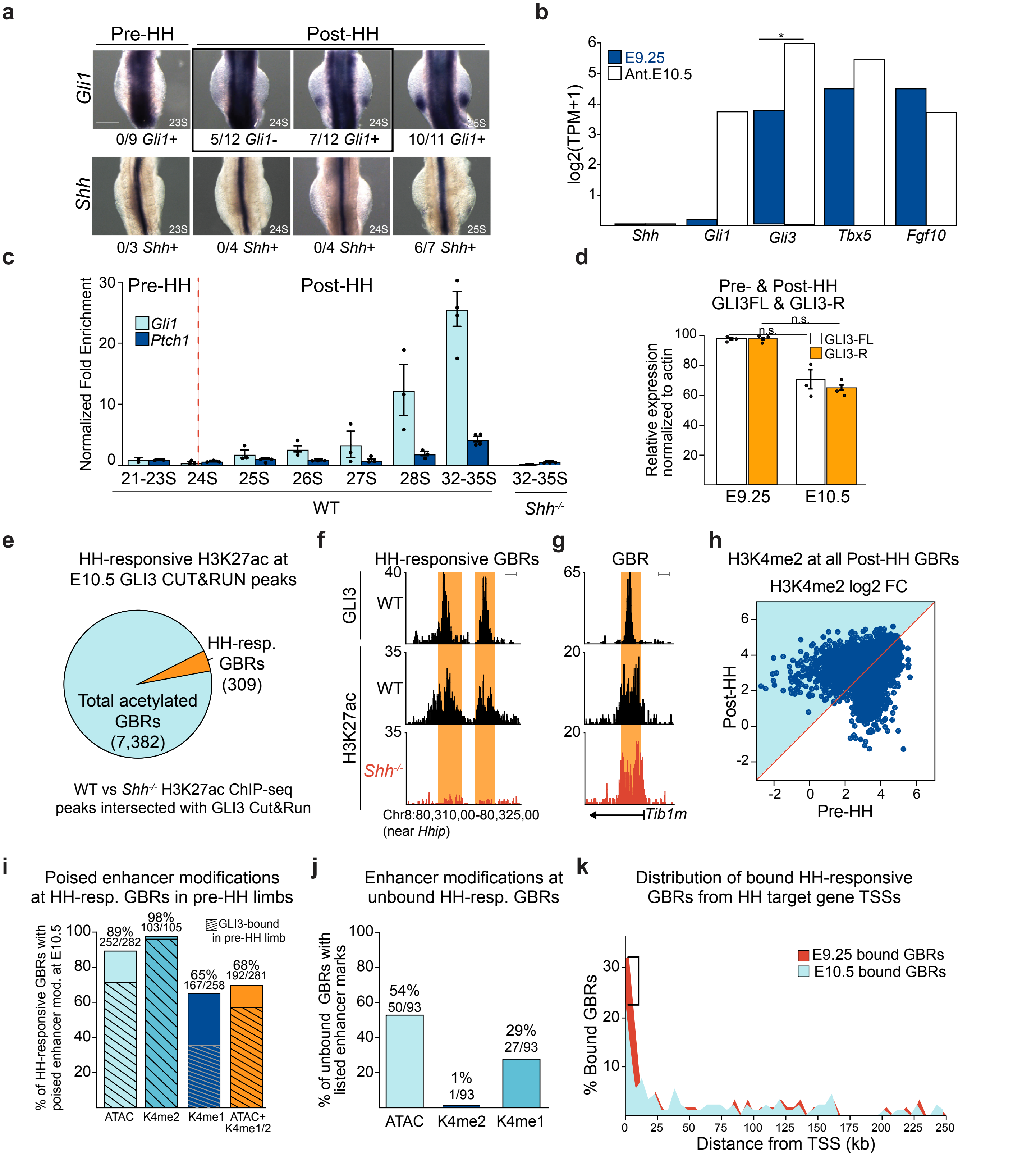

Supplementary Fig.1. Properties of GLI3 binding at enhancers before and after the onset of HH signaling. a Whole mount in situ hybridization for *Gli1* and *Shh* in 23-25S embryos to define the onset of HH induction. While *Shh* is not detectable in the limb until 25S, the earliest detection of *Gli1* is at 24S, where 5/15 embryos have *Gli1* expression. **b** Expression values for *Shh, Gli1* and selected limb genes, in E9.25 and E10.5 anterior limb buds from RNA-seq data. Asterisk indicates adjusted p-value=0.006, two-sided statistical test, multiple hypothesis testing adjusted using BH method. **c** Quantitative PCR showing expression of *Ptch1* and *Gli1* in forelimb pairs from individual embryos

Supplementary Fig.1. cont'd... at specified somite stage. *Ptch1* and *Gli1* expression was normalized to *Gapdh*. RNA was isolated from individual forelimbs using Trizol and cDNA was generated using Superscript IV with ezDNAse (Invitrogen), The *Ptch1*, *Gli1* and *Gapdh* primers were used previously[28]. For 21-23S embryos n=2, for wild-type 32-35S n=4, for all other stages (wild-type 24-28S and *Shh*$^{-/-}$ 32-35S) n=3. All replicates are single pairs of dissected forelimbs from individual embryos (error bars indicate SEM). **d** Quantification of GLI3FL and GLI3-R from representative western blot in Fig.1c and additional western blot replicates in Source Data (n=3 biologically independent samples; error bars indicate SEM). Unpaired, two-sided t-tests were performed. **e** Intersection of E10.5 GLI3 CUT&RUN peaks with previously published WT vs *Shh*$^{-/-}$ E10.5 H3K27ac ChIP-seq peaks[10]. H3K27ac peaks reduced in *Shh*$^{-/-}$ limbs that overlap GLI3 binding regions were categorized as HH-responsive GBRs. **f,g** Examples of a HH-responsive GBR that loses acetylation in *Shh*$^{-/-}$ limbs (**f**) and a GBR that maintains acetylation in the absence of HH signaling (**g**). Orange shading in tracks indicate the GBRs. **h** Scatterplot showing H3K4me2 ChIP-seq fold enrichment at E10.5 GBRs in pre-HH (21-23S) and post-HH (32-35S) limb buds (n=2 biological replicates at each stage). No peaks were significantly changed between pre- and post-HH signaling. **i** Percentage of HH-responsive GBRs enriched (called peaks) for the poised enhancer markers H3K4me1 (CUT&Tag, n=3) and H3K4me2 (n=2) and accessible chromatin (ATAC-seq peaks, n=2) prior to HH signaling. Percentages indicate the number of E9.25 GBRs enriched for the specified feature, out of the total number of HH-responsive GBRs enriched for that enhancer modification at E10.5. **j** Enhancer modifications present at HH-responsive GBRs that are not bound by GLI3 at E9.25. **k** Distribution of E9.25 and E10.5 GBRs from the TSSs of putative HH target genes. Scale bars for

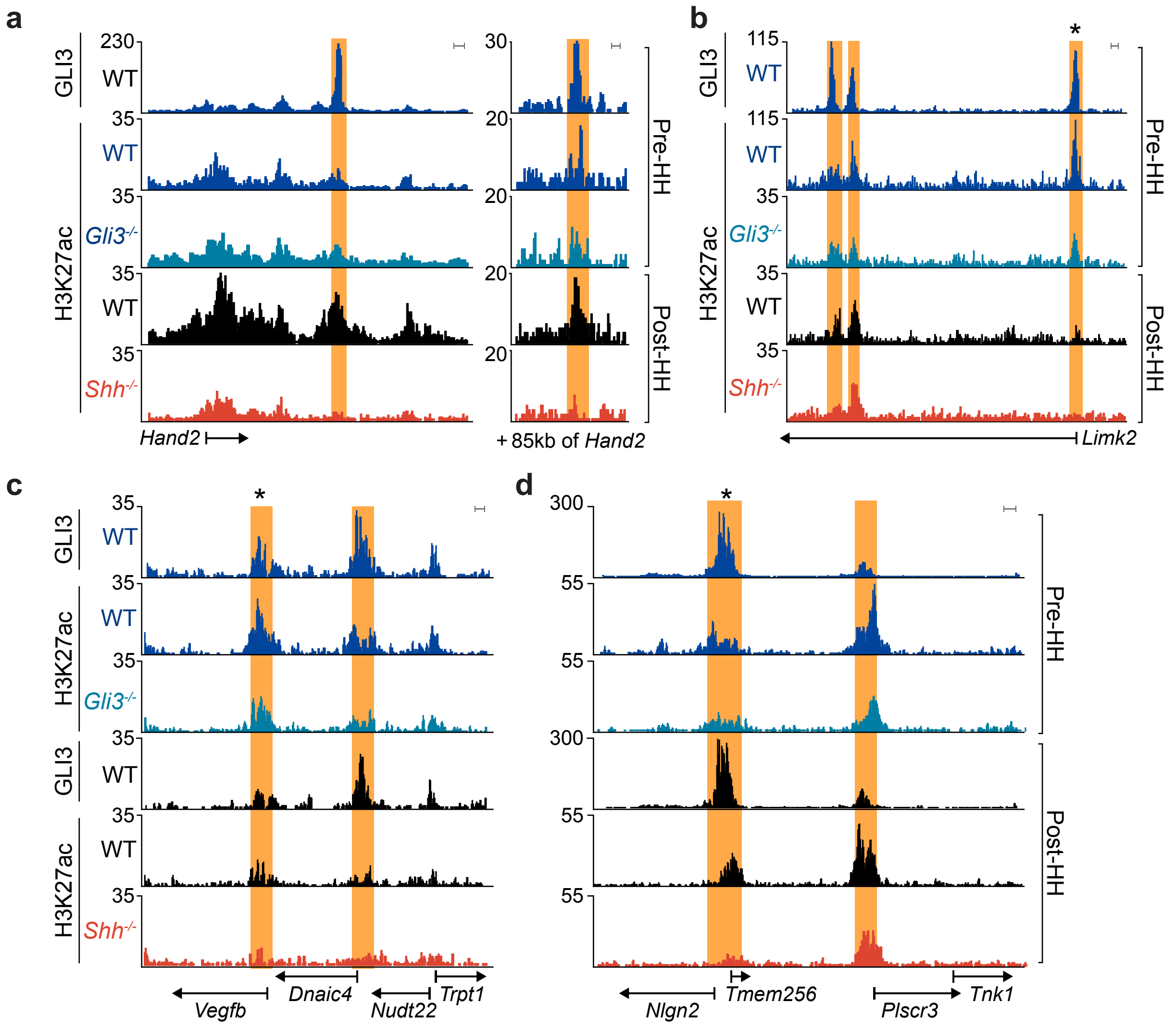

Supplementary Fig.2. E9.25 acetylated GBRs that do not further increase H3K27ac levels with loss of *Gli3*. a HH-responsive GBRs (orange shading) near the GLI3 target Hand2 that have H3K27ac enrichment at E9.25 do not have increased levels of H3K27ac in *Gli3*$^{-/-}$ limb buds, suggesting there is no de-repression of *Hand2*. **b-d** HH-responsive GBRs (orange shading) with significantly higher acetylation prior to HH induction (indicated by asterisk; FDR<0.05), do not have increased acetylation with the absence of *Gli3*. Scale bars for tracks indicate 1kb.

a

Gene	Half-life(hrs)
Gpx6	18.4
She	2.87
Foxd1	3.16
Foxf1	2.58
Osr1	3.02
Mapre3	11.71
Cxcl13	24
Hsd11b2	24
**Average**	**11.2**
**Median**	**7.43**

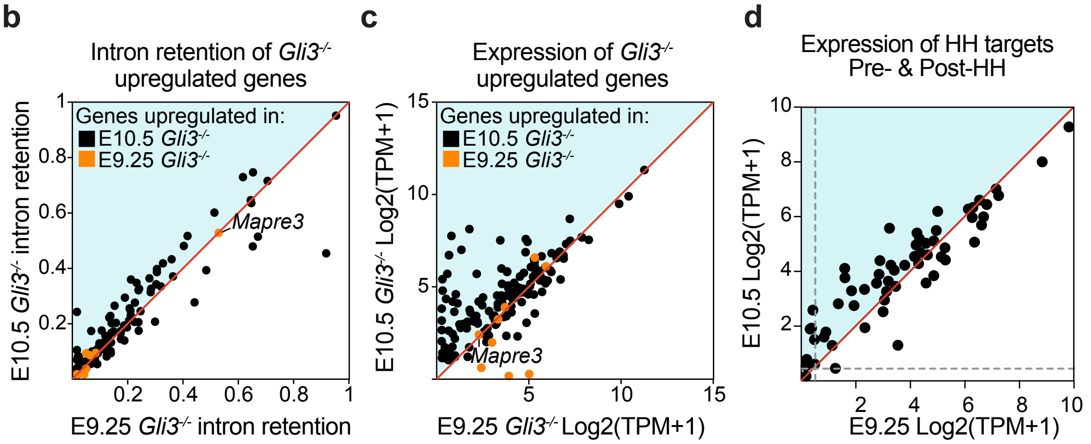

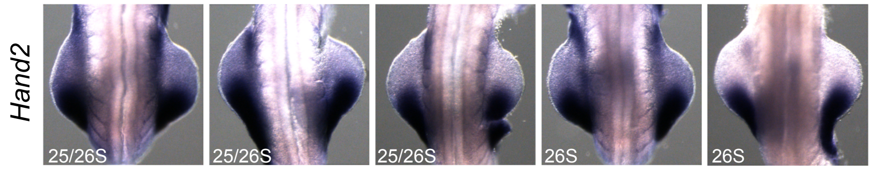

Supplementary Fig.3. E9.25 *Gli3*$^{-/-}$ upregulated genes are expressed at low levels and have low intron retention rates. a Table of half-lives E9.25 Gli3$^{-/-}$ upregulated genes, previously determined in differentiating embryonic stem cells[35]. **b** Intron retention levels in E9.25 and E10.5 *Gli3*$^{-/-}$ upregulated genes. While E10.5 upregulated genes have intron retention rates that vary, E9.25 upregulated genes have intron retention rates near 0, suggestive of mature transcripts. The exception to this is one gene, *Mapre3*, which has high intron retention in both E9.25 and E10.5 *Gli3*$^{-/-}$, but is not upregulated at E10.5. The formula (1-N)/M was used to calculate the intron retention rate for each gene, where N= the number of reads overlapping with any of the gene's exons and M= the number of reads overlapping with the body of the gene. Limma was used to identify differential intron retention rate between E9.25 and E10.5 *Gli3*$^{-/-}$ limbs, FDR cutoff< 0.05. **c** Expression of genes upregulated in E9.25 and E10.5 *Gli3*$^{-/-}$ limb buds. **d** Expression of 74 predicted, direct HH target genes in wild-type limb buds, pre- (E9.25) and post-HH (E10.5). **e** Expression of Hand2 in WT limb buds at ~26S. Note that most embryos have some anterior expression of Hand2 and Hand2 is only completely posteriorly restricted in one embryo at this stage (far right).

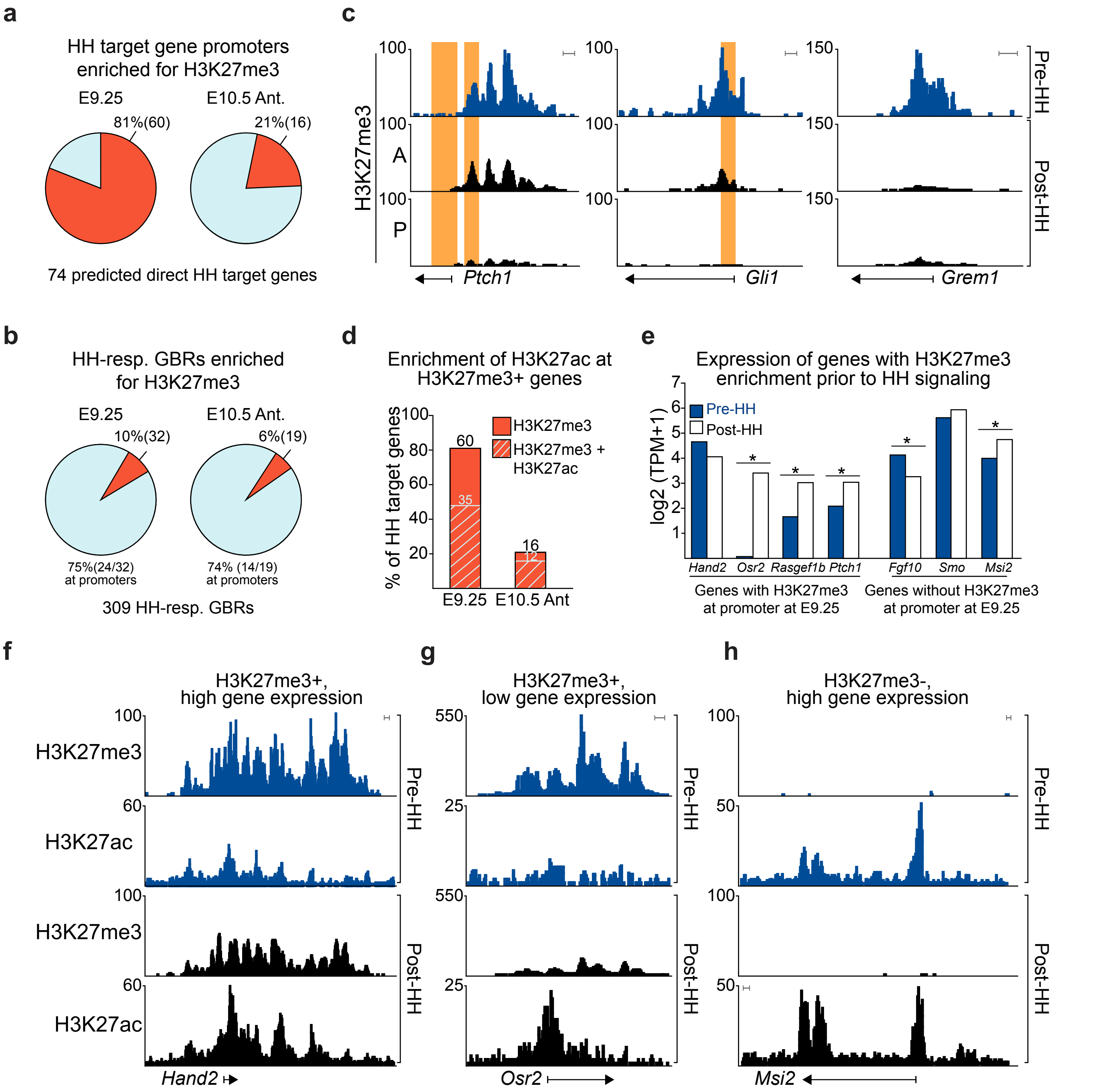

Supplementary Fig.4. H3K27me3 is enriched at many HH target genes prior to HH signaling. **a,b** H3K27me3 enrichment at the promoters of predicted direct HH target gene promoters (**a**) and HH-responsive GBRs (**b**) in E9.25 and E10.5 anterior limb buds. **c** Examples of H3K27me3 enrichment at promoters of HH target genes in E9.25 and anterior and posterior E10.5 limb buds. Orange shading indicates the binding regions for HH-responsive GBRs defined in Figure S1C. **d** Percentage of HH target gene promoters with H3K27me3 and H3K27ac at E9.25 and E10.5. **e** Relative expression levels of genes at E9.25 and E10.5 derived from RNA-seq data (Supplementary Data 3) with and without H3K27me3 enrichment at promoters at E9.25. Asterisks indicate adjusted p-value<0.05, two-sided statistical test, multiple hypothesis testing adjusted using BH method. **f-h** Examples of H3K27me3 and H3K27ac enrichment of genes with high expression at E9.25 with H3K27me3 enrich-ment (**f**), low expression with H3K27me3 enrichment (**g**) and high expression without H3K27me3 enrichment (**h**). Scale bars for tracks indicate 1kb.

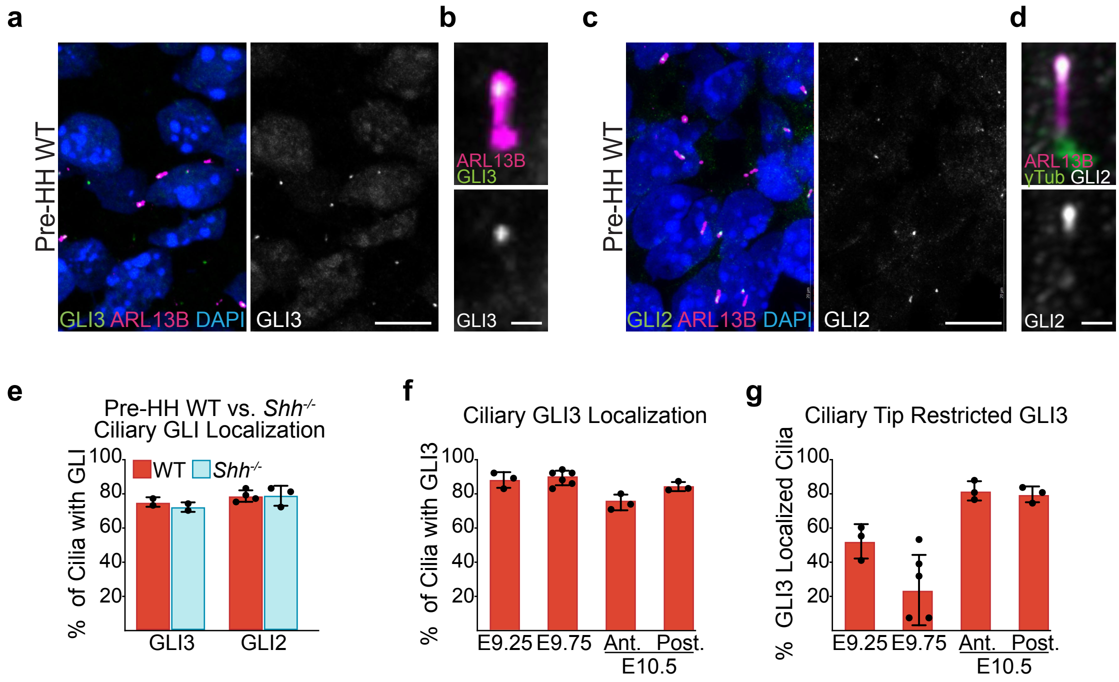

Supplementary Fig.5. GLI ciliary distribution and localization in developing limbs. Images were collected with a Nikon A1R Resonant Scanning Confocal System and are maximum intensity projections. **a,b** Endogenous GLI3^{FLAG} and ARL13b in a representative pre-HH E9.25 WT limb bud, and a representative cilium with GLI3^{FLAG} localization. **c,d** Endogenous GLI2, ARL3b and γ-TUBULIN (basal body) in a representative pre-HH E9.25 WT limb bud. GLI2 is localized to the opposite ciliary end as γ-TUBULIN indicating most GLI localization is at the ciliary tips and not the base (**d**). **e** Quantification of GLI2 and GLI3^{FLAG} ciliary localization in E9.25 WT and *Shh*$^{-/-}$ limb buds (n=2 and 4 biological replicates, respectively). Note the similar levels of GLI2 and GLI3 ciliary localization in both genotypes, suggesting unprocessed GLI proteins are localized at the ciliary tip. **f** Quantifications of GLI3 ciliary localization in pre-HH E9.25 (21-23S; n=3), E9.75 (26-28S n=6) distal limb buds and E10.5 (35S; n=3) anterior and posterior limb buds. **g** Quantification of cilia in the same dataset with GLI3 restricted to the ciliary tip. Error bars in **e-g** indicate SEM. Scale bars for panels **a** and **c** indicate 10μm; scale bards for panels **b** and **d** indicate 0.5μm.

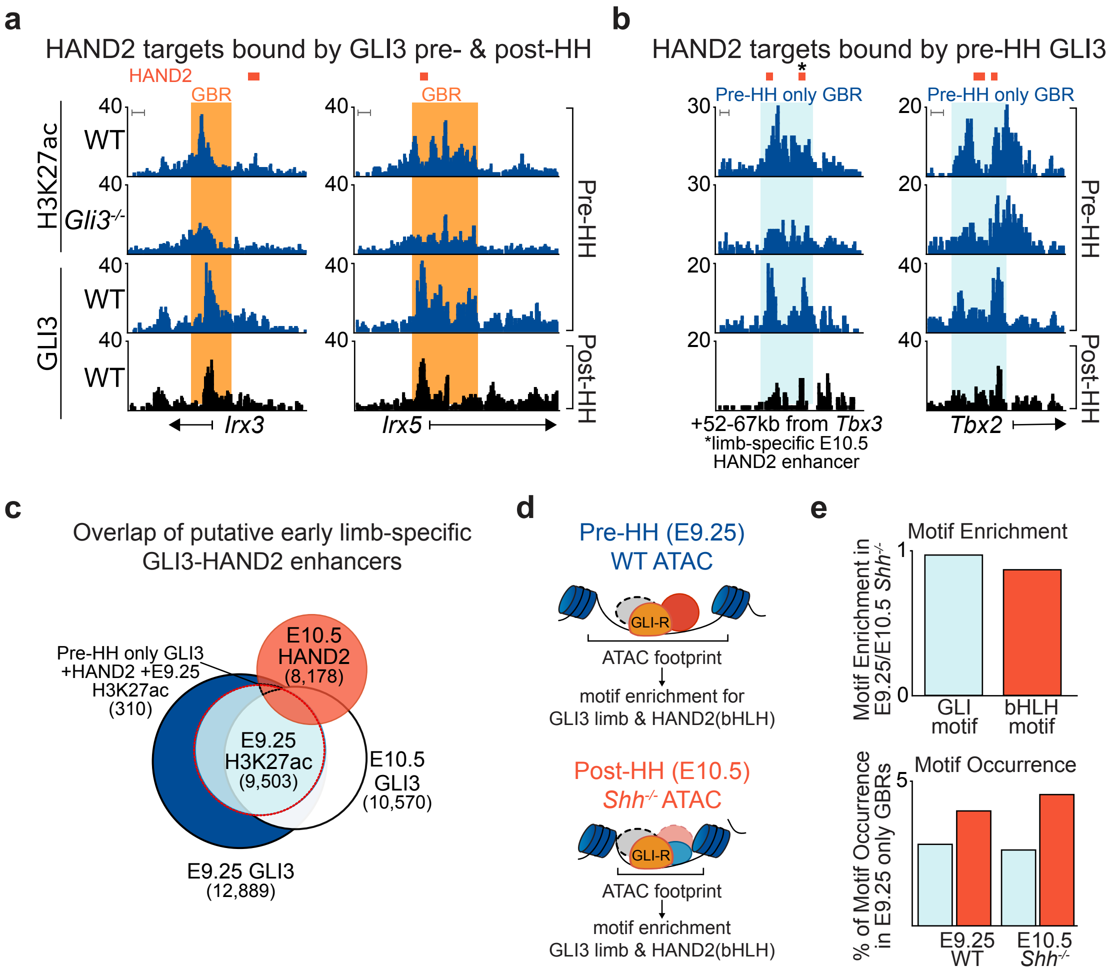

Supplementary Fig.6. Co-localization of HAND2 and GLI3 in pre-HH limb buds. **a** Examples of genes with HAND2 binding regions in E10.5 limbs (red bars denote previously identified *Hand2* regions[14]) that are also bound by GLI3 (orange shading) in pre-HH or post-HH E10.5 limb buds. **b** HAND2 target genes *Tbx2* and *Tbx3* have HAND2 bound regions that overlap with pre-HH specific GBRs (blue shading) but not post-HH GLI3 binding regions. Asterisks denote previously identified HAND2-bound enhancers[14]. **c** Venn diagram of pre- and post-HH (E10.5) GLI3 binding regions, pre-HH H3K27ac peaks and previously identified E10.5 HAND2 limb binding sites[14]. 310 regions are bound by GLI3 only in pre-HH limb buds (not post-HH), overlap with HAND2 binding regions and are acetylated, indicating possibility of these regions being active enhancers. **d** Schematic for performing motif enrichment in ATAC footprints identified in pre-HH WT limbs and compared to ATAC footprints in E10.5 *Shh*$^{-/-}$ limbs. **e** Quantification of enrichment (top) and occurrence (bottom) of limb GLI3 and face HAND2 motifs in the 310 acetylated pre-HH only GBRs overlapping HAND2 binding sites, compared to all acetylated pre-HH GBRs (red circle in **d**), for both pre-HH WT and post-HH E10.5 *Shh*$^{-/-}$ limbs[10,14]. Note that GLI3 and HAND2 motif enrichment and occurrence are unchanged pre-HH WT and post-HH E10.5 *Shh*$^{-/-}$ limbs. Scale bars denote 1kb.

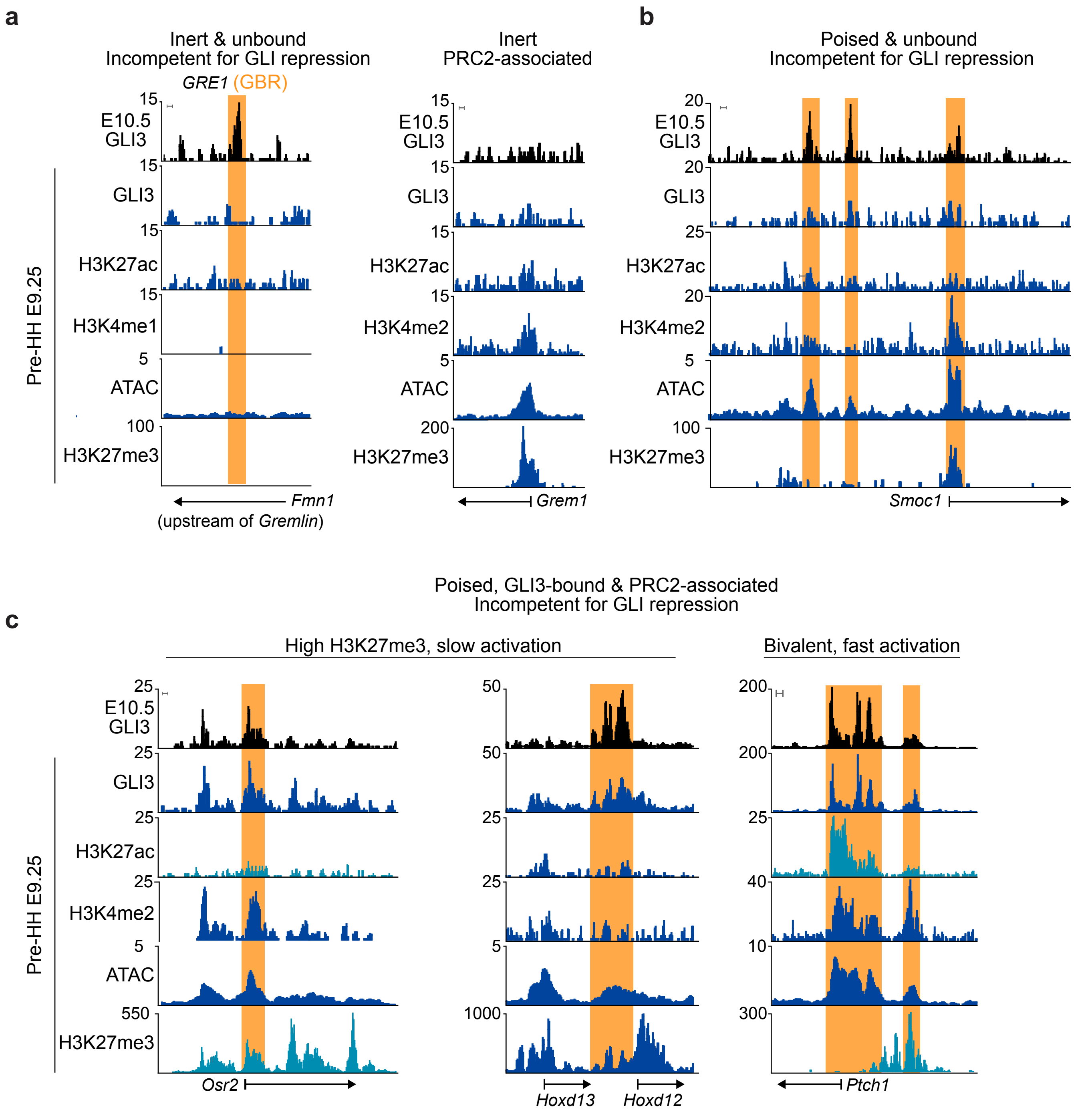

Supplementary Fig.7. GLI3 target genes not competent for GLI repression. **a** Enhancer profiles of the HH target gene Gremlin and GRE1, the GBR regulating Gremlin. GRE1 is not bound by GLI3 prior to HH signaling and is not poised or accessible, while the promoter of Gremlin has high H3K27me3 enrichment, and thus is not competent for GLI repression. **b** Example of GBRs that are not bound but are poised, near HH target Smoc1. bound that are not bound by GLI3 prior to HH signaling at E9.25 and are not competent for GLI3 repression. **c** Examples of GBRs at genes that are bound by GLI3 and poised, but have high H3K27me3 and are likely not competent for GLI3 repression. Differences in H3K27ac at gene promoters may result in slow (left, middle) or fast (right) gene activation. Scale bars =1kb.

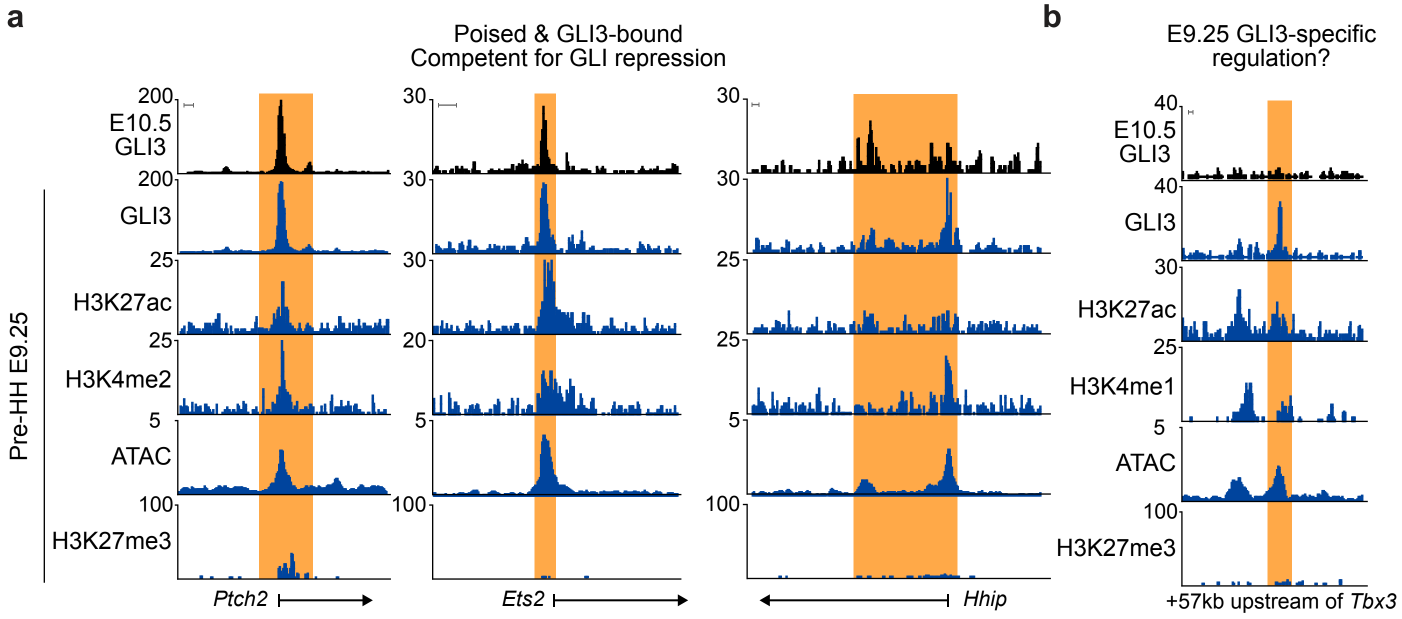

Supplementary Fig.8. GLI3 targets competent for GLI repression. **a** Examples of GBRs bound by GLI3 at E9.25, that are poised and lack H3K27me3 and should be competent for GLI3 repression prior to HH. **b** Example of a GBR that is poised and bound by GLI3 specifically at E9.25, representing a potential stage-specific GLI regulated target. Scale bars =1kb.
