# Supplementary material for: GLI transcriptional repression is inert prior to Hedgehog pathway activation
Source: Nat Commun. 2022 Feb 10;13:808. doi: 10.1038/s41467-022-28485-4 (PMC8831537; doi:10.1038/s41467-022-28485-4)
Supplement: Supplementary file 3 — Description of Supplementary Data Files [file 41467_2022_28485_MOESM3_ESM.pdf]

Description of Additional Supplementary Files

File Name: Supplementary Data 1-5
Description: This folder contains the following 5 data sets:

Supplementary Data 1. All called CUT&RUN (GLI3, HDAC1/2) and CUT&Tag peaks (H3K4me1, H3K27me3).

Supplementary Data 2. All differential ChIP-seq analyses with called peaks (H3K27ac, H3K4me2).

Supplementary Data 3. Differential RNA-seq analyses- WT vs *Gli3*$^{-/-}$; E9.25 vs. E10.5 Anterior limb buds.

Supplementary Data 4. Putative HH target genes and chromatin marks enriched at gene promoters.

Supplementary Data 5. Differential ATAC-seq time course analyses.
