# Supplementary material for: GLI transcriptional repression is inert prior to Hedgehog pathway activation
Source: Nat Commun. 2022 Feb 10;13:808. doi: 10.1038/s41467-022-28485-4 (PMC8831537; doi:10.1038/s41467-022-28485-4)
Supplement: Supplementary file 5 — Reporting Summary [file 41467_2022_28485_MOESM5_ESM.pdf]

nature portfolio

Corresponding author(s): Steven A Vokes

Last updated by author(s): Dec 24, 2021

Reporting Summary

Nature Portfolio wishes to improve the reproducibility of the work that we publish. This form provides structure for consistency and transparency in reporting. For further information on Nature Portfolio policies, see our Editorial Policies and the Editorial Policy Checklist.

Statistics

For all statistical analyses, confirm that the following items are present in the figure legend, table legend, main text, or Methods section.

n/a	Confirmed	
☐	☒	The exact sample size (*n*) for each experimental group/condition, given as a discrete number and unit of measurement
☒	☐	A statement on whether measurements were taken from distinct samples or whether the same sample was measured repeatedly
☐	☒	The statistical test(s) used AND whether they are one- or two-sided<br>*Only common tests should be described solely by name; describe more complex techniques in the Methods section.*
☐	☒	A description of all covariates tested
☐	☒	A description of any assumptions or corrections, such as tests of normality and adjustment for multiple comparisons
☐	☒	A full description of the statistical parameters including central tendency (e.g. means) or other basic estimates (e.g. regression coefficient) AND variation (e.g. standard deviation) or associated estimates of uncertainty (e.g. confidence intervals)
☐	☒	For null hypothesis testing, the test statistic (e.g. F, t, r) with confidence intervals, effect sizes, degrees of freedom and P value noted<br>*Give P values as exact values whenever suitable.*
☒	☐	For Bayesian analysis, information on the choice of priors and Markov chain Monte Carlo settings
☒	☐	For hierarchical and complex designs, identification of the appropriate level for tests and full reporting of outcomes
☒	☐	Estimates of effect sizes (e.g. Cohen's *d*, Pearson's *r*), indicating how they were calculated

Our web collection on statistics for biologists contains articles on many of the points above.

Software and code

Policy information about availability of computer code

Data collection	The pipeline used to identify putative direct HH target genes can be found at https://github.com/Boksunni/Predicted-HH-List-Generation (https://zenodo.org/badge/latestdoi/379668324).
Data analysis	RNA-seq data was analyzed using HISAT2 for alignment and DESeq2 for differential gene expression analysis. CUT&RUN peaks were called using MACS2, ChIP-seq peaks were called using CisGenome version 2.1.0, and CUT&Tag peaks were called using SEACR. Differential peak calling and intron retention were performed with Limma verions 3.14.

For manuscripts utilizing custom algorithms or software that are central to the research but not yet described in published literature, software must be made available to editors and reviewers. We strongly encourage code deposition in a community repository (e.g. GitHub). See the Nature Portfolio guidelines for submitting code & software for further information.

Data

Policy information about availability of data

All manuscripts must include a data availability statement. This statement should provide the following information, where applicable:

- Accession codes, unique identifiers, or web links for publicly available datasets
- A description of any restrictions on data availability
- For clinical datasets or third party data, please ensure that the statement adheres to our policy

All genomic datasets generated in this study, including CUT&RUN, CUT&Tag, ATAC-Seq, ChIP-seq and RNA-seq datasets, were deposited in GEO (GSE178838). Called peaks or differential analyses for these datasets are available in the Source Data. Additional datasets used in this study, include: E10.5 WT vs. Shh-/- H3K27ac ChIP-seq, E10.5 Shh-/- vs. Shh-/-;Gli3-/- microChIP-seq, E10.5 WT H3K4me2 (GSE108880) and E10.5 WT H3K4me1 ChIP-seq (GSE86690), E10.5 WT limb ChIP-seq H3K4me3 (GSE86698) and mouse TAD boundaries (GSE96107).

Field-specific reporting

Please select the one below that is the best fit for your research. If you are not sure, read the appropriate sections before making your selection.

- [x] Life sciences
- [ ] Behavioural & social sciences
- [ ] Ecological, evolutionary & environmental sciences

For a reference copy of the document with all sections, see nature.com/documents/nr-reporting-summary-flat.pdf

Life sciences study design

All studies must disclose on these points even when the disclosure is negative.

Sample size	Sample size was determined based on the cell number requirement of the experiment and the number of cells in a given tissue for each embryonic stage. For instance CUT&Tag required 100,000 cells per replicate while ChIP-seq required 300-600k cells depending on the antibody. Samples from E9.25 embryos yielded ~10,000 cells per pair of limb buds while E10.5 embryos yielded ~100,000 cells per pair of limb buds. Thus, the number of embryos used per experiment was dependent on embryonic stage and cell number input for experiment.
Data exclusions	No data was excluded from analyses.
Replication	2-4 replicates were used for all genomic experiments based on current standards for these techniques. For genomic experiments, successful replicates were ones that passed library QCs via BioAnalyzer traces. All replicates that passed this QC were sequenced and used in this study. On occasion (<10% of total experimental attempts), some libraries would not have optimal material and not pass this QC, a quality we attributed to using low cell-numbers and perhaps having some material loss during initial cell wash steps post-dissection.<br><br>The number of replicates for in situ hybridization ranged from ~3-8 to be able to show variability in expression across different stages. In situ hybridization were used for qualitative but not quantitative conclusions. All attempts to collect in situ replicates were successful (high signal to noise; domain-specific signal) and used in this study.
Randomization	There was no randomization for samples in this study. For embryos used, embryos of the same stage would be pooled from several litters in order to collect sufficient cell numbers for experiments.
Blinding	For GLI3 ciliary quantifications of immunofluorescence images, all samples were quantified blindly. No other experiments required collection or quantification to be blinded, as all other quantifications done were performed computationally on genomic datasets.

Reporting for specific materials, systems and methods

We require information from authors about some types of materials, experimental systems and methods used in many studies. Here, indicate whether each material, system or method listed is relevant to your study. If you are not sure if a list item applies to your research, read the appropriate section before selecting a response.

Materials & experimental systems

n/a	Involved in the study	
[ ]	[x]	Antibodies
[x]	[ ]	Eukaryotic cell lines
[x]	[ ]	Palaeontology and archaeology
[ ]	[x]	Animals and other organisms
[x]	[ ]	Human research participants
[x]	[ ]	Clinical data
[x]	[ ]	Dual use research of concern

Methods

n/a	Involved in the study	
[ ]	[x]	ChIP-seq
[x]	[ ]	Flow cytometry
[x]	[ ]	MRI-based neuroimaging

Antibodies

Antibodies used	M2 FLAG (Sigma-Aldrich; F3165), Arl13b (Proteintech; 17711-1-AP), GLI2 (a gift from Jonathon Eggenschwiler), Gamma tubulin (Sigma #T6557), B-actin (Cell Signaling #8457), H3K27ac (Abcam #ab4729) , H3K4me2 (Millipore #07-030), H3K4me1 (Abcam #ab8895), H3K27me3 (abcam #ab195477), HDAC1 (Abcam ab7028), HDAC2 (Abcam ab7029), Goat anti-mouse Alexa568 (Thermo Fisher Scientific; A-11004) and Goat anti-rabbit Alexa488 (Thermo Fisher Scientific; A-11034), Donkey anti-guinea pig Alexa594 (Jackson ImmunoResearch 706-585-148), Donkey anti-mouse (Jackson #715-035-150)and Donkey anti-rabbit (Jackson #711-005-0152).
Validation	Antibody dilutions for western blots were specified by manufacturer. Antibody dilutions and conditions for immunofluoresence experiments were validated by ensuring high signal to noise, integrity of tissue nuclei, and that signals were reproducible across sections within an embryo and between embryos. All antibodies used for CUT&RUN (FLAG/GLI3, HDAC1/2) and CUT&Tag (H3K4me1, H3K27me3) were tested on E10.5 limb bud tissue. Test samples that passed library QCs by BioAnalyzer traces that were consistent with manufacturer's example library traces, were then sequenced to validate optimal antibody concentrations. Validation was determined by identifying antibody dilutions with high signal to noise on bigwig tracks that were similar to previously generated ChIP-

seq signals for that antibody and with >50% overlap in ChIP-seq called peaks.

Animals and other organisms

Policy information about studies involving animals; ARRIVE guidelines recommended for reporting animal research

Laboratory animals	Animals used: Mus musculus. The Gli3Xt-J (Jackson Cat# 000026) and Shh-tm1amc null (Jackson Cat# 003318) alleles were maintained on a Swiss Webster background. The Gli3-3XFLAG allele, with an N-terminal 3XFLAG-epitope was maintained on a mixed background. Generally, females >6 weeks of age were used for breeding and embryos were collected between E9.25-E10.5 (21-35S). All experiments were performed in accordance with IACUC guidelines.
Wild animals	No wild animals were used for this study.
Field-collected samples	No field-collected samples were used for this study.
Ethics oversight	Experiments involving mice were approved by the Institutional Animal Care and Use Committee at the University of Texas at Austin (protocol AUP-2019-00233).

Note that full information on the approval of the study protocol must also be provided in the manuscript.

ChIP-seq

Data deposition

☒ Confirm that both raw and final processed data have been deposited in a public database such as GEO.

☒ Confirm that you have deposited or provided access to graph files (e.g. BED files) for the called peaks.

Data access links
May remain private before publication.

All of our datasets have been uploaded to GEO (GSE178838) and are publicly available.

Files in database submission

E9_Gli3_rep1.bigwig E9_Gli3_rep1_S16_R1.fastq.gz E9_Gli3_rep1_S16_R2.fastq.gz
E9_Gli3_rep2.bigwig E9_Gli3_rep2_S17_R1.fastq.gz E9_Gli3_rep2_S17_R2.fastq.gz
E10_Gli3_rep1.bigwig E10_Gli3_rep1_S18_R1.fastq.gz E10_Gli3_rep1_S18_R2.fastq.gz
Gli3_250s.bigwig Gli3-250s_S29_R1.fastq.gz Gli3-250s_S29_R2.fastq.gz
E9_me1_14.bigwig E9-me1-14_S24_R1.fastq.gz E9-me1-14_S24_R2.fastq.gz
E9_me1_6.bigwig E9-me1-6_S25_R1.fastq.gz E9-me1-6_S25_R2.fastq.gz
E9_H3K4me1_CnT_rep3.bigwig E9-CnT-me1_S1_L001_R1_001.fastq.gz E9-CnT-me1_S1_L001_R2_001.fastq.gz
me2_sample1.bigwig E9_me2_rep1_S56_R1.fastq.gz
me2_sample2.bigwig E9_me2_rep2_S57_R1.fastq.gz
input_sample1.bigwig E9_inpt_rep1_S54_R1.fastq.gz
9-HDAC1.bam.bw 9-HDAC1_R1.fastq.gz 9-HDAC1_R2.fastq.gz
9-HDAC2-1.bam.bw 9-HDAC2-1_R1.fastq.gz 9-HDAC2-1_R2.fastq.gz
9-HDAC2.bam.bw 9-HDAC2_R1.fastq.gz 9-HDAC2_R2.fastq.gz
10-HDAC1-1.bam.bw 10-HDAC1-1_R1.fastq.gz 10-HDAC1-1_R2.fastq.gz
10-HDAC1-2.bam.bw 10-HDAC1-2_R1.fastq.gz 10-HDAC1-2_R2.fastq.gz
10-HDAC1-3.bam.bw 10-HDAC1-3_R1.fastq.gz 10-HDAC1-3_R2.fastq.gz
10-HDAC2-1.bam.bw 10-HDAC2-1_R1.fastq.gz 10-HDAC2-1_R2.fastq.gz
10-HDAC2-2.bam.bw 10-HDAC2-2_R1.fastq.gz 10-HDAC2-2_R2.fastq.gz
10-HDAC2-3.bam.bw 10-HDAC2-3_R1.fastq.gz 10-HDAC2-3_R2.fastq.gz
E9-WT-1.bigwig E9-WT-1_S1_R1.fastq.gz
E9-WT-2.bigwig E9-WT-2_S4_R1.fastq.gz
WT-input.bigwig WT-input_S6_R1.fastq.gz
E9-Gli3-1.bigwig E9-Gli3-1_S2_R1.fastq.gz
E9-Gli3-2.bigwig E9-Gli3-2_S3_R1.fastq.gz
Gli3-input.bigwig Gli3-input_S5_R1.fastq.gz
E9WT1_S12_L001_R1_001.fastq.gz.out E9WT1_S12_L001_R1_001.fastq.gz
E9WT2_S13_L001_R1_001.fastq.gz.out E9WT2_S13_L001_R1_001.fastq.gz
E9WT3_S14_L001_R1_001.fastq.gz.out E9WT3_S14_L001_R1_001.fastq.gz
E9Gli31_S8_L001_R1_001.fastq.gz.out E9Gli31_S8_L001_R1_001.fastq.gz
E9Gli32_S9_L001_R1_001.fastq.gz.out E9Gli32_S9_L001_R1_001.fastq.gz
E9Gli33_S10_L001_R1_001.fastq.gz.out E9Gli33_S10_L001_R1_001.fastq.gz
E9Gli34_S11_L001_R1_001.fastq.gz.out E9Gli34_S11_L001_R1_001.fastq.gz
E10WT1_S4_L001_R1_001.fastq.gz.out E10WT1_S4_L001_R1_001.fastq.gz
E10WT2_S5_L001_R1_001.fastq.gz.out E10WT2_S5_L001_R1_001.fastq.gz
E10WT3_S6_L001_R1_001.fastq.gz.out E10WT3_S6_L001_R1_001.fastq.gz
E10WT4_S7_L001_R1_001.fastq.gz.out E10WT4_S7_L001_R1_001.fastq.gz
E10Gli32_S1_L001_R1_001.fastq.gz.out E10Gli32_S1_L001_R1_001.fastq.gz
E10Gli33_S2_L001_R1_001.fastq.gz.out E10Gli33_S2_L001_R1_001.fastq.gz
E10Gli34_S3_L001_R1_001.fastq.gz.out E10Gli34_S3_L001_R1_001.fastq.gz
E9_me3_16.bigwig E9-me3-16_S26_R1.fastq.gz E9-me3-16_S26_R2.fastq.gz

E9_me3_2.bigwig E9-me3-2_S27_R1.fastq.gz E9-me3-2_S27_R2.fastq.gz
10A1-m3-6.bam.bw 10A1-m3-6_R1.fastq.gz 10A1-m3-6_R2.fastq.gz
10P1-m3-7.bam.bw 10P1-m3-7_R1.fastq.gz 10P1-m3-7_R2.fastq.gz
A1-K27me3.bam.bw A1-K27me3_R1.fastq.gz A1-K27me3_R2.fastq.gz
P1-K27me3.bam.bw P1-K27me3_R1.fastq.gz P1-K27me3_R2.fastq.gz
A2-K27me3.bam.bw A2-K27me3_R1.fastq.gz A2-K27me3_R2.fastq.gz
P2-K27me3.bam.bw P2-K27me3_R1.fastq.gz P2-K27me3_R2.fastq.gz
E9-15.bam.bw E9-15_S2_L001_R1_001.fastq.gz E9-15_S2_L001_R2_001.fastq.gz
E9-18.bam.bw E9-18_S3_L001_R1_001.fastq.gz E9-18_S3_L001_R2_001.fastq.gz
WT-28S-1.bam.bw WT-28S-1_S12_L001_R1_001.fastq.gz WT-28S-1_S12_L001_R2_001.fastq.gz
WT-28S-2.bam.bw WT-28S-2_S13_L001_R1_001.fastq.gz WT-28S-2_S13_L001_R2_001.fastq.gz
WT-28S-3.bam.bw WT-28S-3_S14_L001_R1_001.fastq.gz WT-28S-3_S14_L001_R2_001.fastq.gz
ShhE19_S31.bam.bw ShhE19_S31_L002_R1_001.fastq.gz
ShhE20_S32.bam.bw ShhE20_S32_L002_R1_001.fastq.gz
ShhE22_S34.bam.bw ShhE22_S34_L002_R1_001.fastq.gz
E10-16.bam.bw E10-16_S1_L001_R1_001.fastq.gz E10-16_S1_L001_R2_001.fastq.gz
WT-35S-1.bam.bw WT-35S-1_S16_L001_R1_001.fastq.gz WT-35S-1_S16_L001_R2_001.fastq.gz
WT-35S-2.bam.bw WT-35S-2_S17_L001_R1_001.fastq.gz WT-35S-2_S17_L001_R2_001.fastq.gz
ShhL11_S35.bam.bw ShhL11_S35_L002_R1_001.fastq.gz
ShhL9_S38.bam.bw ShhL9_S38_L002_R1_001.fastq.gz
Shh-L11.bam.bw Shh-L9_S11_L001_R2_001.fastq.gz Shh-L9_S11_L001_R1_001.fastq.gz
Shh-L9.bam.bw Shh-L11_S10_L001_R2_001.fastq.gz Shh-L11_S10_L001_R1_001.fastq.gz
S-nul-G684.bam.bw S-nul-G684_S5_L001_R2_001.fastq.gz S-nul-G684_S5_L001_R1_001.fastq.gz
S-het-G685.bam.bw S-het-G685_S4_L001_R2_001.fastq.gz S-het-G685_S4_L001_R1_001.fastq.gz
SG-1.bam.bw SG-1_S6_L001_R2_001.fastq.gz SG-1_S6_L001_R1_001.fastq.gz
SG-2.bam.bw SG-2_S7_L001_R2_001.fastq.gz SG-2_S7_L001_R1_001.fastq.gz
SG-3.bam.bw SG-3_S8_L001_R2_001.fastq.gz SG-3_S8_L001_R1_001.fastq.gz

Genome browser session (e.g. UCSC)

Bigwigs are provided in the GEO accession but not available on Genome browser.

Methodology

Replicates

Two replicates were performed for ChIP-seq experiments. For other similar genomic chromatin binding, histone modification etc. (ATAC-seq, CUT&RUN, CUT&Tag) 2-4 replicates were used.

Sequencing depth

For ChIP-seq experiments, a minimum of 30 million single end reads were sequenced per sample. For other genomic experiments- ATAC-seq: ~>50 million PE or SE reads; CUT&Tag/RUN: 3-5 million PE reads were sequenced.

Antibodies

M2 FLAG (Sigma-Aldrich; F3165), H3K27ac (Abcam #ab4729) , H3K4me2 (Millipore #07-030), H3K4me1 (Abcam #ab8895), H3K27me3 (abcam #ab195477), HDAC1 (Abcam ab7028) and HDAC2 (Abcam ab7029).

Peak calling parameters

macs2 callpeak -g mm -f BAM --nomodel -t $1 -n $2 --nolambda --keep-dup all -B

Data quality

PCR duplicates and unpaired reads were removed. For peak calling an FDR cutoff <0.05 was used.

Software

MACS was used for ChIP-seq and CUT&RUN peak calling.
